# New mitogenomic evidence for the phylogeny of Siphonaptera: insights from *Callopsylla dolabris* and *Oropsylla silantiewi*

**DOI:** 10.3389/fvets.2026.1876060

**Published:** 2026-08-04

**Authors:** Wendi Zhang, Jun Wu, Jiao Luo, Mingna Duan, Shaobo Tang, Yujia Huang, Wei Gu, Dandan Jiang, Xing Yang

**Affiliations:** 1 Integrated Laboratory of Pathogenic Biology, College of Preclinical Medicine, Dali University, Dali, Yunnan, China; 2 Department of Ophthalmology, Dali Bai Autonomous Prefecture People's Hospital, Dali, Yunnan, China; 3 Department of Infection, The First Affiliated Hospital of Dali University, Dali, Yunnan, China; 4 The Key Laboratory of Infectious Diseases of Yunnan Provincial Education Department, Dali, Yunnan, China; 5 The Clinical Medical Sub-Center of Infectious Diseases of Yunnan Province, Dali, Yunnan, China; 6 School of Public Health, Dali University, Dali, Yunnan, China

**Keywords:** fleas, mitochondrial genome, phylogeny, plague, Siphonaptera

## Abstract

As important vectors of infectious diseases such as plague, fleas require accurate classification and phylogenetic analysis, which are essential for public health prevention and control. However, complete mitochondrial genome resources for Siphonaptera in public databases are still extremely scarce. In this study, the complete mitochondrial genomes of *C. dolabris* and *O. silantiewi*, collected from the high-altitude region in Qinghai Province, were sequenced and characterized for the first time using next-generation sequencing technology. The results showed the two mitogenomes were 17,104 bp and 15,914 bp in length, respectively. Both mitogenomes exhibited significant A+T bias and a strong preference for A/U-ending codons. Selective pressure analysis (Ka/Ks) indicated that mitochondrial protein-coding genes (PCGs) of fleas are predominantly under purifying selection. Phylogenetic reconstructions based on 39 mitogenomic sequences from 36 flea species revealed that Ceratophylloidea was robustly supported as monophyletic. At the family level, neither Ceratophyllidae nor Leptopsyllidae formed a strictly monophyletic group in the present dataset. Pulicidae was recovered as a well-supported monophyletic clade. Both newly sequenced species were placed within Ceratophyllidae. This study not only expands the available mitogenomic resources for Siphonaptera, but also provides new insights into nucleotide composition patterns and phylogenetic relationships within the order.

## 1 Introduction

Flea (Siphonaptera) is a type of obligate, blood-sucking ectoparasitic insect that widely inhabits rodents, carnivores, and some birds, and can transmit various important zoonotic pathogens (1). Among them, plague caused by *Yersinia pestis* remains one of the most severe flea-borne diseases (2). Despite remarkable risk reduction for large plague outbreaks brought by modern public health systems, multiple natural plague foci still exist across all continents. In China, the natural plague focus of *Marmota himalayana* in Qinghai-Tibet Plateau represents one of the most important endemic regions (3, 4). Fleas serve as the main vectors of *Y. pestis*, and their vector competence, host specificity, and ecological adaptation vary considerably among species (5). During plague epizootics, flea community composition and population dynamics are closely related to the severity of animal plague epidemics (6). Therefore, accurate species identification and a better understanding of flea phylogenetic relationships are essential not only for improving basic vector biology, but also for clarifying interactions among pathogens, hosts, and vectors within natural plague foci (7, 8).

*Callopsylla dolabris* (Jordan & Rothschild, 1911) and *Oropsylla silantiewi* (Wagner, 1898) are dominant flea species in the Qinghai-Tibet Plateau plague focus in China. Both are common parasites of *M. himalayana*, and account for 90% of the total parasitic fleas in this area (9). Previous studies have shown that both species exhibit relatively high natural infection rates and strong potential for plague transmission based on epidemiological surveys, host association analyses, and ultrastructural studies of the proventriculus, indicating their important roles as vectors in local plague cycles (10).

However, research on these two fleas still mainly focuses on morphology, host surveys, and epidemiological monitoring (8, 11–14). Molecular genetic data remain limited, particularly complete mitochondrial genome information, which has to some extent limited detailed genetic characterization and comparative genomic and phylogenetic analyses. At present, flea identification relies primarily on external morphology and characters such as male and female genitalia, following established taxonomic keys (15–18). However, these diagnostic traits often require considerable taxonomic expertise, and the availability of reliable characters may differ between sexes. Identification becomes particularly challenging when specimens are damaged or key diagnostic features are missing (19). Therefore, molecular evidence, particularly complete mitochondrial genomes, may serve as a complementary source of information for morphology-based taxonomy and has been applied in species identification, comparative genomics, and phylogenetic inference (20). Due to its relatively conserved structure, maternal inheritance, rapid evolutionary rate, and absence of recombination, mitochondrial genome has become one of the most widely used molecular markers in insect molecular taxonomy, population genetics, and phylogenetic studies (21). Compared with single-gene markers, complete mitochondrial genomes generally contain more phylogenetic information and can improve the resolution of evolutionary analyses (22, 23).

In recent years, high-throughput sequencing has substantially increased the number of available insect mitochondrial genomes, providing valuable resources for evolutionary and comparative genomic studies. Nevertheless, although the number of flea mitochondrial genomes has increased, it remains limited relative to the more than 2,500 described flea species (18, 21), particularly for medically and veterinary relevant vectors. This limited availability of flea mitogenomic resources restricts a more comprehensive understanding of flea phylogeny and mitochondrial genome evolution. Expanding genomic sampling across flea lineages is therefore needed to improve current datasets and support future evolutionary studies.

In this study, we sequenced and characterized the complete mitochondrial genomes of *C. dolabris* and *O. silantiewi*. We analyzed gene content, nucleotide composition, codon usage bias, tRNA secondary structures, and selection pressures. Phylogenetic analyses were conducted using newly generated and publicly available flea mitochondrial genomes to infer the evolutionary placement of the two species within Siphonaptera. This study expands mitochondrial genomic resources for fleas and provides data that may support vector surveillance and studies of host–vector–pathogen interactions.

## 2 Materials and methods

### 2.1 Sample collection and mitochondrial gene extraction

All specimens used in this study were collected from *M. himalayana* in Jiuzhi County, Qinghai Province, China (33°12′N, 101°04′E) at an elevation of approximately 4,000 m. The sample set consisted of 51 individuals of *C. dolabris* (36 females and 15 males) and 53 individuals of *O. silantiewi* (40 females and 13 males). Specimen identification was performed by specialists in Siphonaptera taxonomy under an Olympus stereomicroscope based on key diagnostic morphological characters, including the frontal tubercle, pronotal comb, and male and female genital structures, following the taxonomic keys and species descriptions provided in *Fauna Sinica, Insecta: Siphonaptera* (2nd Edition, 2007) (17). Voucher specimens were deposited in the Dali University Parasitological Museum under voucher numbers DLUP2712 (*C. dolabris*) and DLUP2716 (*O. silantiewi*). The remaining validated specimens were stored at −20 °C for subsequent use.

The total genomic DNA of fleas was extracted using a modified CTAB method, and no mitochondrial DNA enrichment was performed. Extracted DNA was used for subsequent library preparation and high-throughput sequencing. All animal procedures in this study were performed in strict compliance with institutional ethical standards and were formally approved by the Laboratory Animal Management Committee of Dali University (Approval No. 2023-P2-280).

### 2.2 Mitochondrial gene assembly and annotation

Whole-genome shotgun libraries with an insert size of approximately 400 bp were constructed using the KAPA DNA HyperPrep Kit (Roche, USA) and sequenced on an Illumina NovaSeq platform with a paired-end 150 bp strategy. A total of 22,047,636 raw reads were generated for *C. dolabris* and 23,333,258 for *O. silantiewi*, corresponding to 3.33 Gb and 3.52 Gb of raw data, respectively. The Q20 values were 98.56% and 99.00%, and the Q30 values were 94.93% and 96.11%. Raw reads were processed using fastp (24) to remove adapter sequences and low-quality reads, yielding clean reads of 21,417,140 (*C. dolabris*) and 22,702,908 (*O. silantiewi*). All raw sequencing data have been deposited in the Sequence Read Archive (SRA) under accession number PRJNA1483469. The average sequencing depths of the mitochondrial genomes were 916.16 × and 1,341.39 × , respectively, with coverage statistics shown in Supplementary Figures S1, S2. Clean reads were first assembled *de novo* using SPAdes v3.15.4 (25) and GetOrganelle v1.7.7 (26) with default parameters, generating candidate contigs. Putative mitochondrial sequences were then identified based on assembly results and verified through BLASTn (27) searches against the NCBI nt database. The assembly graphs were subsequently examined using Bandage v0.8.1 (28) to determine the connectivity and relative positions of mitochondrial contigs. Where gaps were present between adjacent contigs, the terminal regions were extended using the original sequencing reads to reconstruct a circular mitochondrial genome assembly. The assembled genome was subsequently polished using Pilon v1.18 (29) to generate the final mitochondrial genome sequence. The complete assembled mitochondrial genomes were deposited in GenBank under accession numbers PX993261 (*C. dolabris*) and PX979559 (*O. silantiewi*). Genome annotation was performed using the MITOS Web Server (30) under the invertebrate mitochondrial genetic code with default parameters. Protein-coding genes, rRNA genes, and tRNA boundaries were manually inspected and corrected where necessary based on the invertebrate mitochondrial genetic code and comparison with homologous flea mitogenomes. Circular mitochondrial genome maps were generated using CGView (31).

### 2.3 Analysis of mitochondrial genome characteristics

The codon usage patterns of the (PCGs) from the two newly sequenced fleas and previously published mitochondrial genomes were calculated using PhyloSuite v2 (32, 33). Codon usage scatter plot was generated using OriginPro 2021. Relative synonymous codon usage (RSCU) diagrams and amino acid frequency plot were generated using R 4.5.3 (34–39). For evolutionary rate analysis, each of the 13 PCGs from 36 flea species was individually aligned using MAFFT v7 (40) implemented in PhyloSuite v2 under the auto strategy (L-INS-i selected), with default parameters. The non-synonymous substitution rate (Ka), synonymous substitution rate (Ks), and the Ka/Ks ratio for each PCG were subsequently calculated based on the NG method using KaKs_Calculator v3 (41). Finally, the nucleotide diversity (Pi) among the 36 Siphonaptera mitochondrial genomes was estimated via a sliding window analysis (window size: 200 bp, step size: 25 bp) in DnaSP v6 (42). The visualization of Ka/Ks ratio and the nucleotide diversity was performed in R 4.5.3 (34–39).

### 2.4 Phylogenetic analysis

Phylogenetic analyses were conducted using both Maximum Likelihood (ML) and Bayesian Inference (BI) methods, respectively, implemented in IQ-TREE v3.0.1 (43) and MrBayes v3.2.7 (44) via PhyloSuite v2. A total of 40 mitochondrial genomes were included, including 39 ingroup mitogenomes representing 36 flea species and one outgroup species (*Boreus elegans*; Supplementary Table S1). To proceed, we pruned the ambiguous and gap-rich regions of the 13 PCGs using Gblocks v0.91b (45) under default settings implemented in PhyloSuite v2. Subsequently, the resulting alignments were concatenated into an 11,048 bp supermatrix for subsequent phylogenetic reconstruction. The optimal partition schemes and best models were inferred by ModelFinder (46), with independent models assigned to each partition. For the ML analysis, node support was evaluated using 1,000 bootstrap replicates. For the BI analysis, two independent Markov Chain Monte Carlo (MCMC) runs were performed. Each run consisted of four chains (three heated and one cold) running for 2 million generations, with tree sampling every 1,000 generations. The posterior probabilities (PP) were calculated after removing the initial 25% of the burn-in sample. Convergence was confirmed using the average standard deviation of split frequencies (ASDSF), indicating adequate mixing across runs (ASDSF < 0.01, PSRF ≈ 1.0, ESS > 300 for all parameters; Supplementary Table S2). Trace plots further supported stationarity and convergence of likelihood and parameter estimates (Supplementary Figures S3–S5). Finally, the phylogenetic trees were presented after being beautified on iTOL website (https://itol.embl.de/). The analysis files are provided in the Supplementary material.

## 3 Results

### 3.1 Mitochondrial genome structure and composition

**Figure 1:**
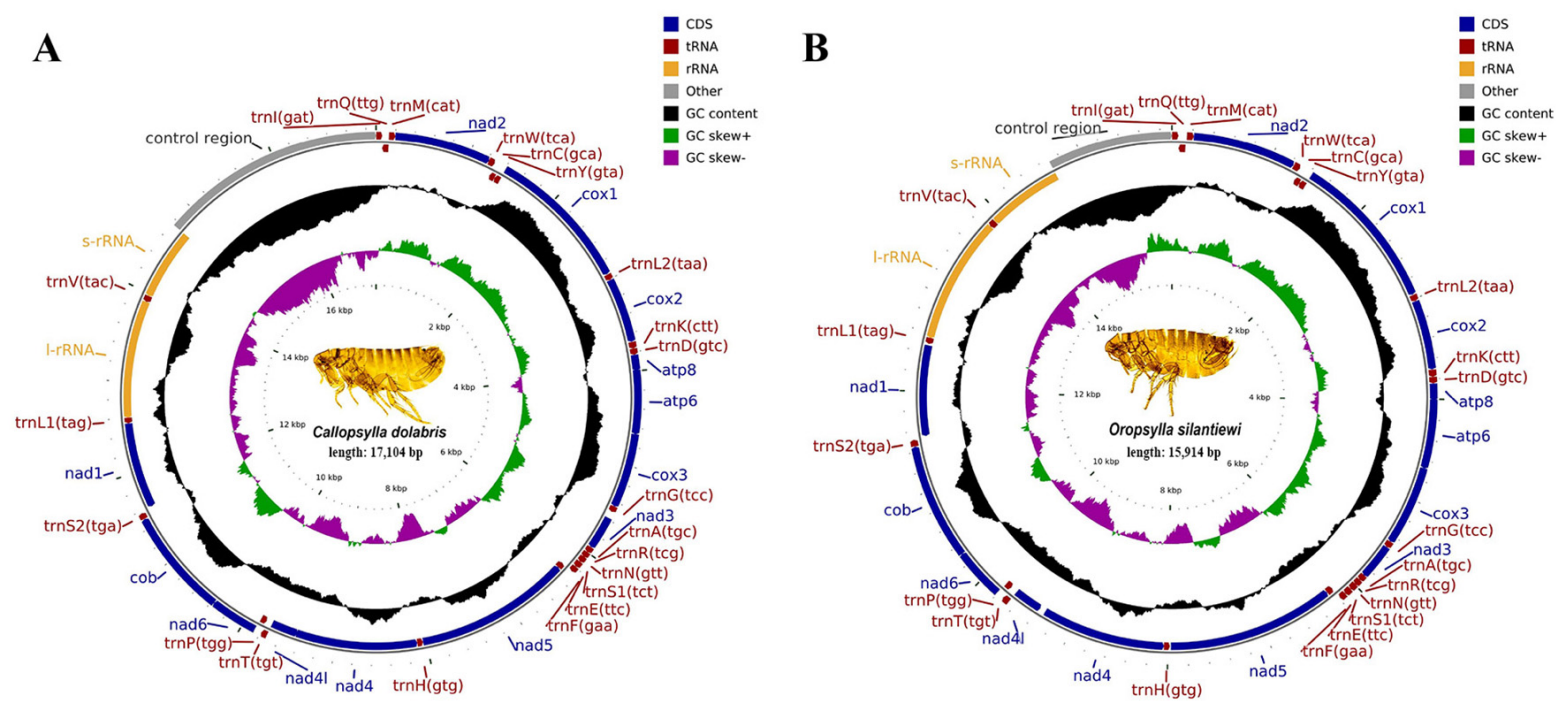
Mitochondrial genome maps of **(A)** *C. dolabris* and **(B)** *O. silantiewi*.

In this study, the newly sequenced and first publicly released mitochondrial genomes of *C. dolabris* and *O. silantiewi* were determined to be 17,104 bp and 15,914 bp in length, respectively. Both genomes comprised the typical 37 genes (13 protein-coding genes, 22 tRNA genes, and two rRNA genes) along with an A + T-rich region (the non-coding control region; Figures 1A, B). Among the annotated genomic features of the two fleas, the shortest element in both mitogenomes was *trnC* (61 bp). The control region (OH) represented the longest annotated element in *C. dolabris* (2,356 bp). In both species, the longest protein-coding gene was *nad5*, measuring 1,717 bp in *C. dolabris* and 1,713 bp in *O. silantiewi*. In terms of intergenic spacers, the longest fragment in *C. dolabris* was 71 bp, located between the *trnG* and *nad3* genes; in *O. silantiewi*, the longest spacer was 59 bp, situated between the *nad4* and *nad4L* genes. In contrast, the longest overlapping region in both mitogenomes was conserved, identically located between the *atp8* and *atp6* genes with a 7-bp overlap (Supplementary Table S3).

The overall nucleotide compositions of the complete mitochondrial genomes for *C. dolabris* and *O. silantiewi* were as follows: A (38.6% and 38.9%), T (41.4% and 39.5%), G (7.8% and 8.2%), and C (12.3% and 13.4%), respectively. Both genomes exhibited a strong A+T bias, with the A + T contents reaching 80.0% and 78.4%, respectively. Furthermore, both newly sequenced species displayed negative values for AT-skew (−0.035 and −0.007) and GC-skew (−0.221 and −0.244; Supplementary Table S4).

**Figure 2:**
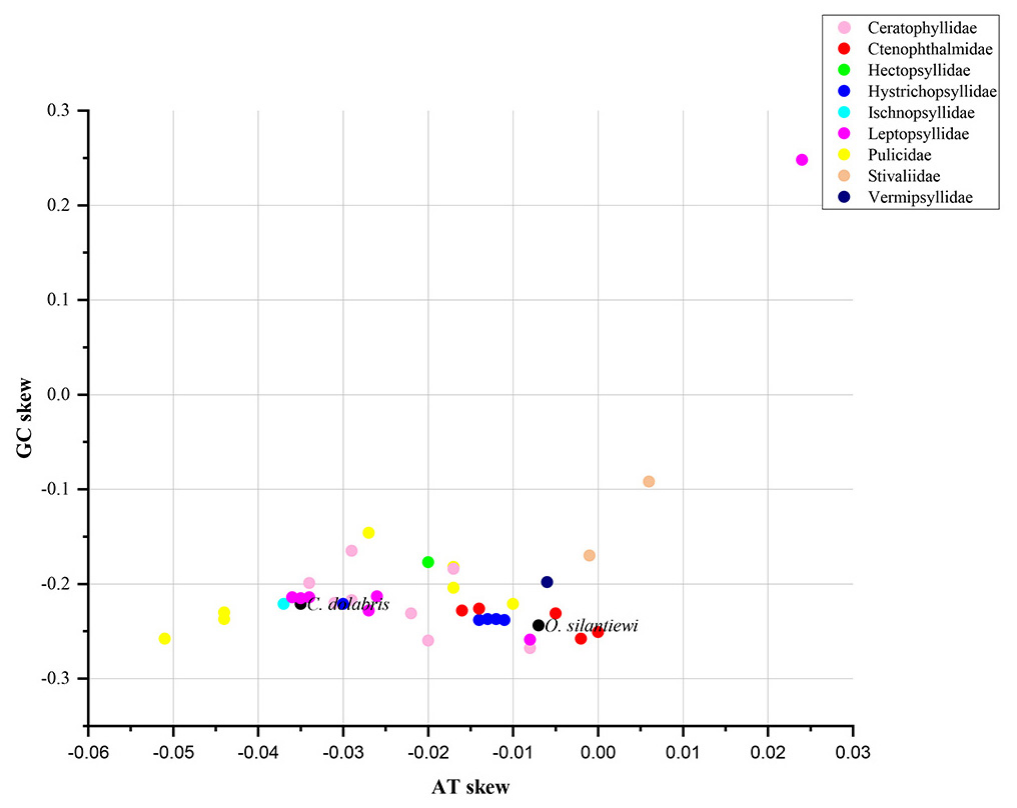
AT and GC skews of flea mitochondrial genomes. *C. dolabris* and *O. silantiewi* are highlighted in black with labels shown on the right.

As illustrated in the scatter plot depicting the nucleotide skewness across 39 Siphonaptera mtDNA sequences (Figure 2), the majority exhibited negative values for both AT-skew and GC-skew. Only two species differed, with *Neopsylla specialis* presenting an AT-skew of zero, and *Leptopsylla segnis* uniquely displaying positive values for both AT-skew (0.024) and GC-skew (0.248) (47).

### 3.2 Protein-coding genes and codon usage

The total lengths of PCGs in the mitogenomes of the two fleas were 11,142 bp and 11,055 bp, with A + T contents of 76.9% and 76.3%, respectively, (Supplementary Table S4). Among the 13 PCGs, *nad1, nad4, nad4L*, and *nad5* were encoded on the Minority strand (N-strand), while the remaining nine genes were located on the Majority strand (J-strand; Supplementary Table S3). The nucleotide composition of individual PCGs varied notably across genes and between the two species. Specifically, in *C. dolabris*, the highest A+T content was observed in *nad6* (84.4%) and the lowest in *cox1* (70.3%). In *O. silantiewi*, the highest A+T content was found in *atp8* (86.1%), with the lowest also in *cox1* (68.7%). Moreover, all PCGs exhibited a negative AT-skew. Notably, the four N-strand genes (*nad1, nad4, nad4L*, and *nad5*) consistently displayed a negative AT-skew coupled with a positive GC-skew in both species (Supplementary Table S4).

The usage patterns of start and stop codons were generally conserved and exhibited high consistency between the two species. With regard to initiation codons, except for *nad5* (ATG/ATT), *nad6* (ATC/ATT), and *nad1* (ATG/ATA), the remaining genes used the same initiation signals in both species, mainly ATT, ATC, and ATG. Regarding termination codons, except for *nad3* (TAA/TAG), *nad5* [T(AA)/TAA], and *nad1* (TAG/TAA), all remaining PCGs shared identical termination codons, using either the complete stop codon TAA or an incomplete stop codon T(AA; Supplementary Table S3). The incomplete stop codon T(AA) is presumed to be completed to TAA by post-transcriptional polyadenylation (48).

**Figure 3:**
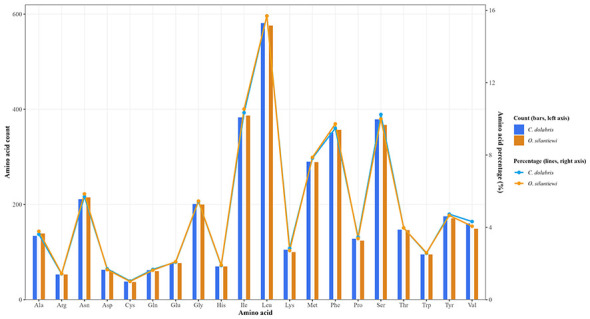
Amino acid composition of mitochondrial protein-coding genes (PCGs) in *C. dolabris* and *O. silantiewi*. Blue and orange indicate the two species, respectively.

When it comes to amino acid usage bias, the trend of *C. dolabris and O. silantiewi* was the same, with Leucine (Leu) accounting for the highest proportion, while Cysteine (Cys) had the lowest. Among the 20 standard amino acids, PCGs strongly preferred to encode Leu (15.69% and 15.69%), Ile (10.34% and 10.54%), Ser (10.24% and 9.99%), and Phe (9.48% and 9.72%). Conversely, Cys was rarely used, with only 38 and 37 occurrences, accounting for 1.03% and 1.01%, respectively (Figure 3).

### 3.3 Transfer and ribosomal RNA genes

There was a total of 22 tRNA genes in the mitochondrial genomes of the two fleas, among which *trnQ, trnC, trnY, trnF, trnH, trnP, trnL1*, and *trnV* were located on the N-strand (Supplementary Table S3). The tRNA sequences of *C. dolabris* and *O. silantiewi* exhibited positive values for both AT-skew (0.017 and 0.028) and GC-skew (0.128 and 0.140; Supplementary Table S4).

Secondary structure predictions revealed that all tRNAs except *trnS1* could fold into the classic cloverleaf secondary structure which consists of four core domains called the amino acid acceptor stem, the DHU arm, the anticodon arm, and the TΨC arm. The *trnS1* gene obviously lacked the DHU arm, rendering its cloverleaf structure incomplete. Within the stem regions, Watson-Crick base pairs were predominant, with a small number of G-U wobble pairs. Overall, the tRNA secondary structures were highly conserved between the two species (Supplementary Figure S6).

The two rRNA genes, *rrnS* and *rrnL*, were encoded on N-strand and separated by the intervening *trnV* gene (Supplementary Table S3). Additionally, these sequences also exhibited positive AT-skew and GC-skew.

### 3.4 Codon usage bias analysis

**Figure 4:**
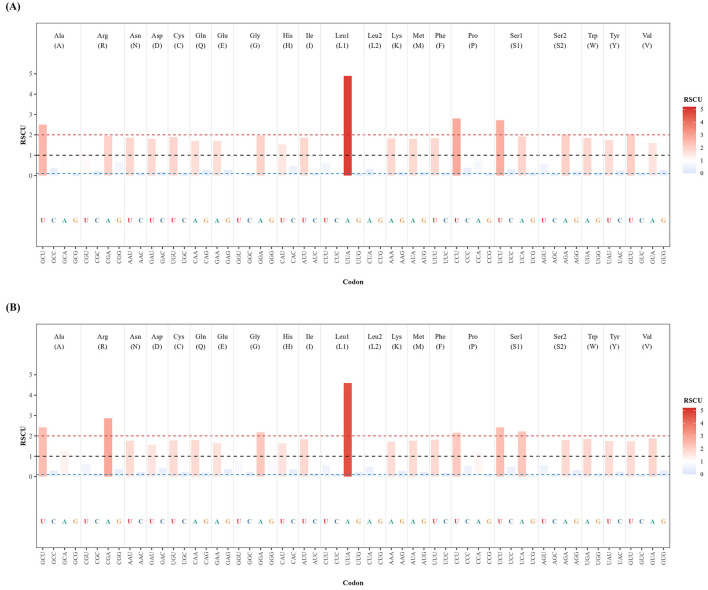
Relative synonymous codon usage (RSCU) of *C. dolabris* **(A)** and *O. silantiewi* **(B)**. Red, black, and blue dashed horizontal lines indicate RSCU values of 2, 1, and 0.1, respectively. Colored letters above codons indicate the terminal base of each codon.

The Relative Synonymous Codon Usage (RSCU) was calculated for the 13 PCGs of the two species, revealing significant codon usage bias in each mitogenome (Figure 4). Codons with RSCU > 1 were defined as preferred, while those with RSCU < 1 were considered unpreferred. In *C. dolabris*, 26 preferred codons were identified. Of these, six displayed strong usage bias: UUA (Leu1), CCU (Pro), UCU (Ser1), GCU (Ala), GUU (Val), and AGA (Ser2). In contrast, 36 codons were unpreferred, of which eight exhibited RSCU values substantially below 1: CUC (Leu1), CUG (Leu2), ACG (Thr), AGC (Ser2), GCG (Ala), CCG (Pro), GGC (Gly), and GUC (Val).

In *O. silantiewi*, 26 preferred codons were also observed. Eight of these showed obvious bias namely UUA (Leu1), CGA (Arg), UCU (Ser1), GCU (Ala), UCA (Ser1), GGA (Gly), CCU (Pro), ACU (Thr). Within the 36 unpreferred codons, five were notably underrepresented, namely CUG (Leu2), GCG (Ala), UCG (Ser1), ACG (Thr), and GUC (Val).

A comparative analysis of the RSCU values revealed a shared preference across the two species for codons ending with A or U, whereas those ending with G or C were notably less frequent. Specifically, the codon UUA was highly favored in *C. dolabris* and *O. silantiewi*, exhibiting the maximum RSCU values of 4.90 and 4.60, respectively. These figures substantially exceeded the RSCU values of the second most frequently used codons in each species, namely CCU (2.81) and CGA (2.86).

### 3.5 The evolutionary rate of genes and sliding window analysis

**Figure 5:**
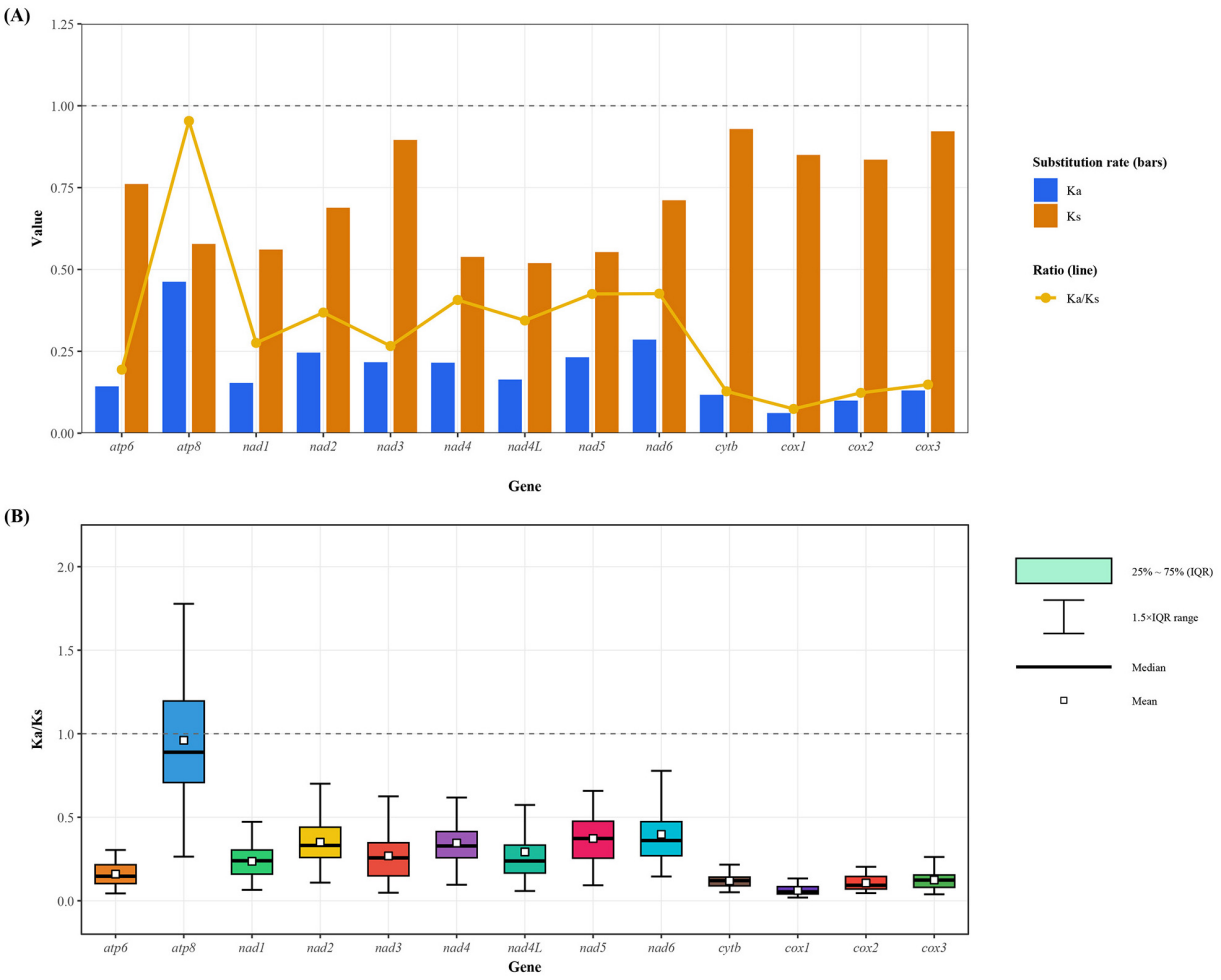
Evolutionary rates of 13 PCGs. **(A)** Average non-synonymous (Ka) rates, synonymous (Ks) rates, and Ka/Ks ratios. Blue and orange bars represent Ka and Ks values, respectively. The yellow line shows the Ka/Ks ratio. **(B)** Boxplots showing the Ka/Ks ratio distribution for each PCG across 36 species. The middle line represents the median, and the open square represents the mean. The box encompasses the 25th to 75th percentiles, while the whiskers represent the range within 1.5 times the interquartile range (IQR). This pattern reflects the overall evolutionary trend across PCGs and should not be interpreted as indicating identical selective pressures acting on every individual gene.

To evaluate evolutionary pressures, the ratios of non-synonymous (Ka) to synonymous (Ks) substitution rates (Ka/Ks) were calculated for the 13 aligned PCGs across 36 analyzed Siphonaptera species. The results revealed that the mean and median Ka/Ks values for all genes were less than 1, suggesting that the mitochondrial amino acid sequences evolved predominantly under purifying selection and remained relatively conserved (Figures 5A, B).

Notably, *atp8* exhibited the highest Ka/Ks values among all analyzed genes, with mean and median Ka/Ks values of 0.95 and 0.83, respectively, and showed substantially greater dispersion than the remaining PCGs. This pattern may reflect relatively relaxed functional constraints acting on *atp8* or reduced stability of Ka/Ks estimation for this comparatively short gene. Other genes exhibited more concentrated distribution patterns under typical purifying selection. Evolutionary rate heterogeneity was also prominently observed. Among the analyzed PCGs, *cox1* evolved the slowest (mean and median = 0.07), suggesting that it was most strongly influenced by purifying selection (Figure 5A).

**Figure 6:**
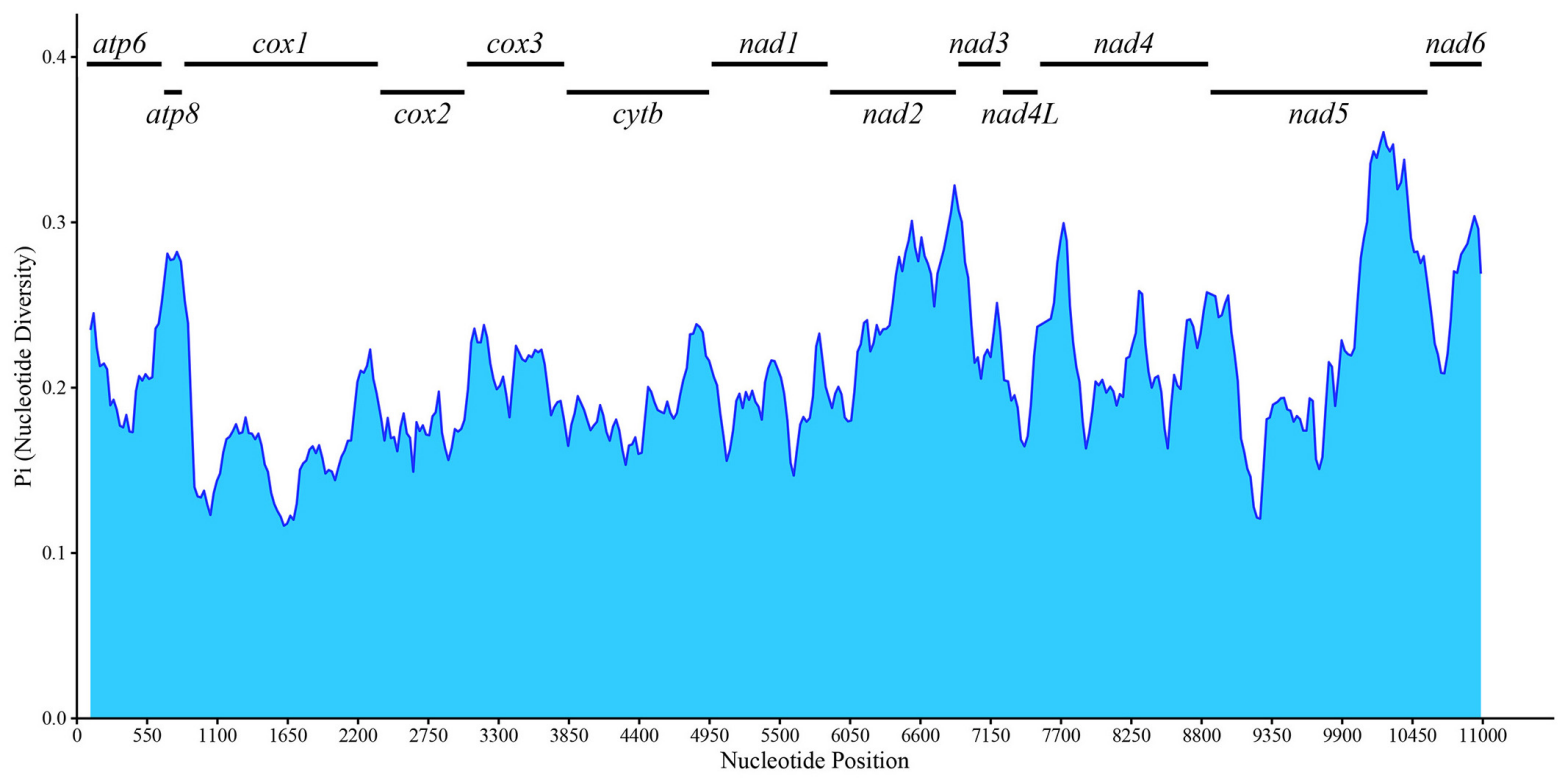
Sliding window analysis of nucleotide diversity (Pi) across 13 mitochondrial protein-coding genes (PCGs) in fleas, with a window size of 200 bp and a step size of 25 bp.

Sliding window analysis of nucleotide diversity (Pi) for 13 PCGs of the 36 Siphonaptera mitogenomes revealed considerable variation in evolutionary rates, demonstrating a significant heterogeneity distribution of sequence variability (Figure 6). Notably, the *nad5* gene region exhibited the highest Pi value, representing the most rapidly evolving segment of the entire mtDNA. Similarly, the *nad6* gene interval and the *nad2* and *nad3* gene intervals also displayed markedly elevated variability. In contrast, the highly conserved regions (i.e., Pi troughs) were mainly centered around *cox1*, which maintained consistently low Pi values and profound sequence conservation.

### 3.6 Phylogenetic analysis

**Figure 7:**
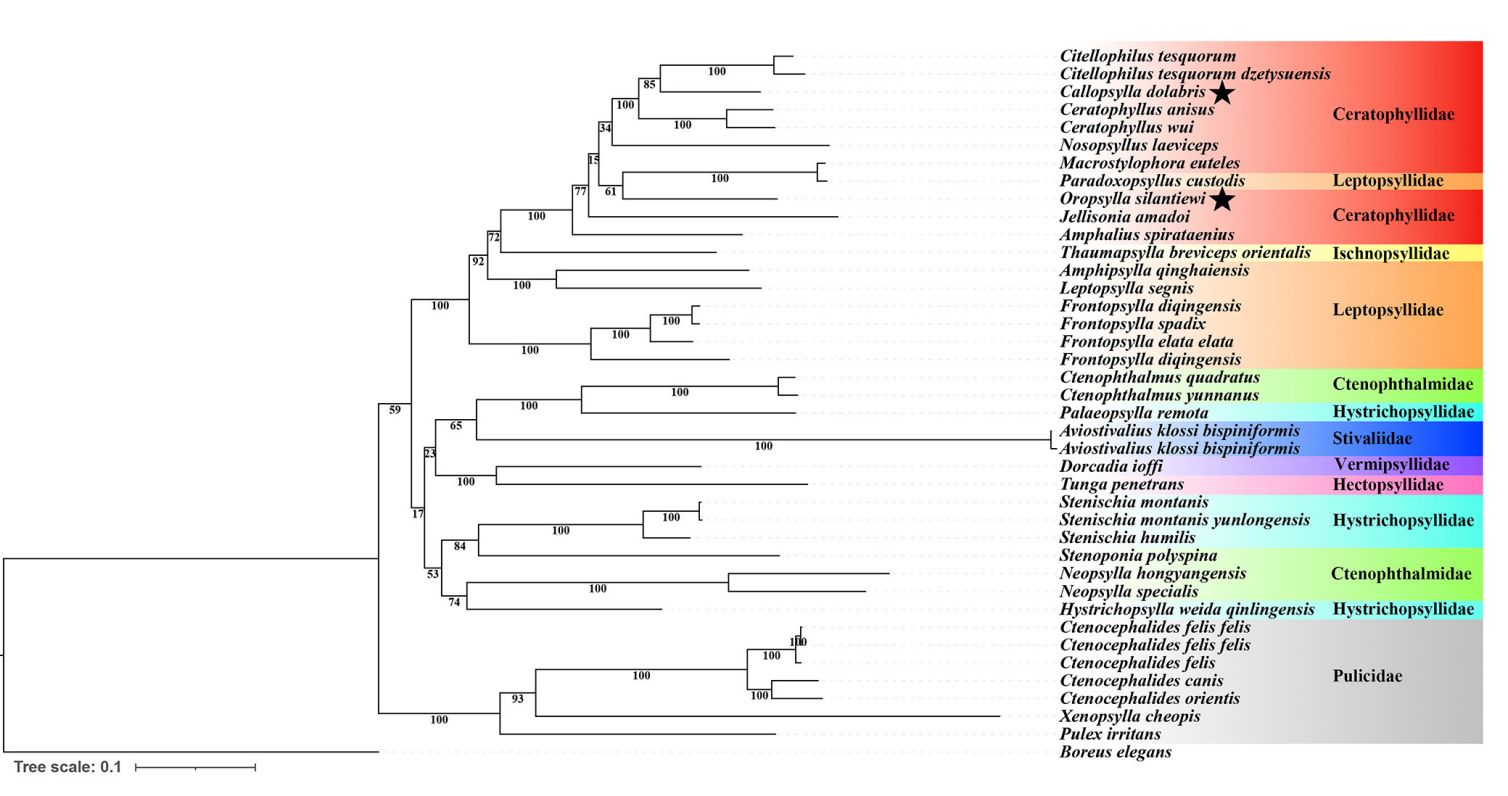
Maximum likelihood (ML) phylogenetic tree based on 39 mitochondrial sequences from 36 flea species. Numbers on branches represent bootstrap support values (BP). Asterisks (⋆) mark the newly sequenced *C. dolabris* and *O. silantiewi*. Color-coded blocks on the right correspond to different taxonomic families, with family names labeled inside each block. Vertical bars and labels on the outermost side denote superfamily-level taxonomic ranks.

**Figure 8:**
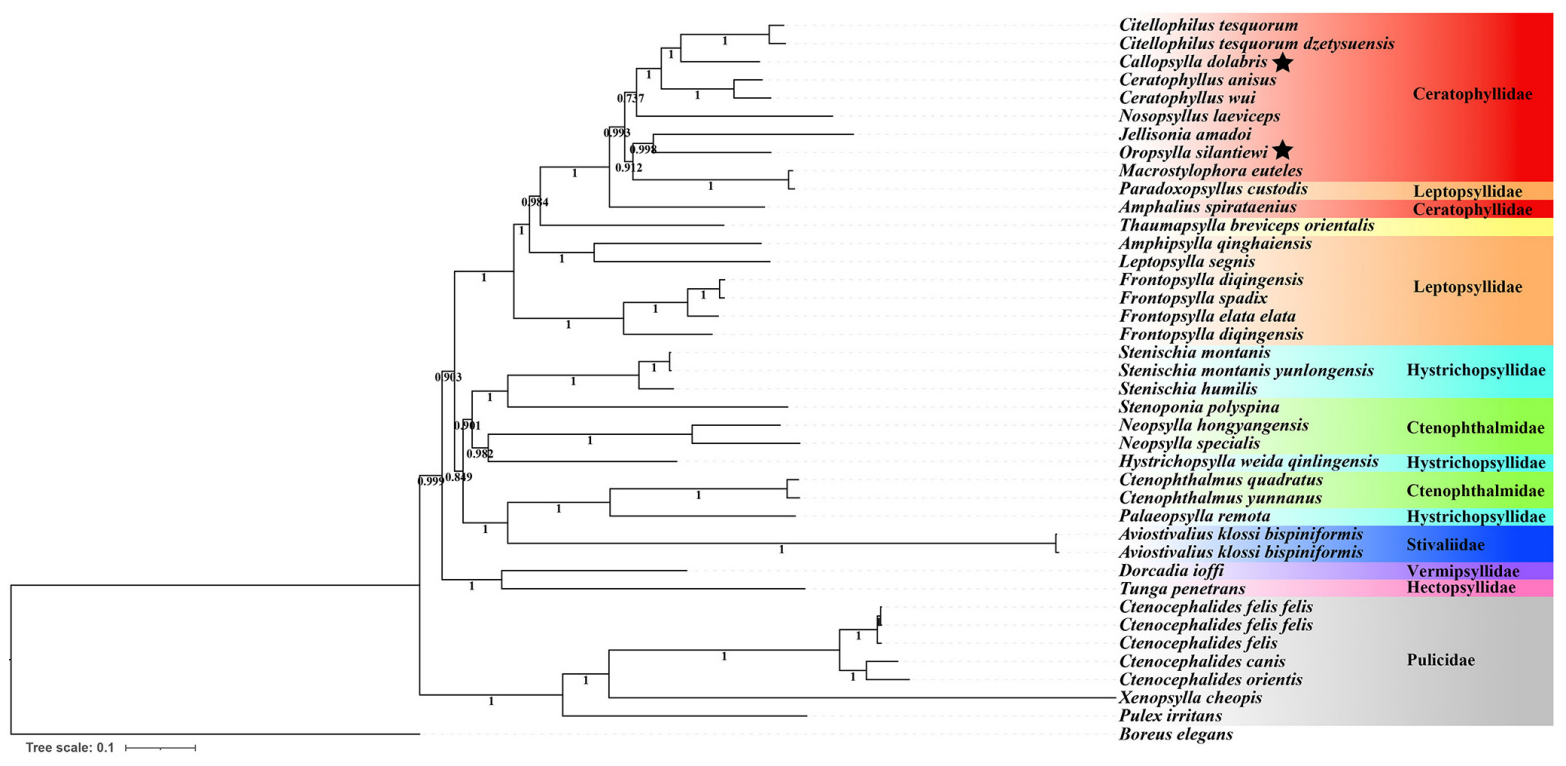
Bayesian Inference (BI) phylogenetic tree based on 39 mitochondrial sequences from 36 flea species. Numbers on branches indicate posterior probabilities (PP). Asterisks (⋆) mark the newly sequenced *C. dolabris* and *O. silantiewi*. Color-coded blocks on the right correspond to different taxonomic families, with family names labeled inside each block. Vertical bars and labels on the outermost side denote superfamily-level taxonomic ranks.

Bayesian inference (BI) and maximum likelihood (ML) phylogenetic trees were reconstructed based on the concatenated sequences of the 13 PCGs from 39 mitogenomes (Table 1), representing 36 species across nine families of Siphonaptera, with *B. elegans* as the outgroup (Figures 7, 8).

**Table 1:** Datasets.

Species	Accession no.
*Amphalius spirataenius*	OR855715
*Amphipsylla qinghaiensis*	PQ571081
*Aviostivalius klossi bispiniformis*	OR774970
*Aviostivalius klossi bispiniformis*	PP963728
*Callopsylla dolabris*	PX993261
*Ceratophyllus anisus*	NC_073017
*Ceratophyllus wui*	NC_040301
*Citellophilus tesquorum*	PP418872
*Citellophilus tesquorum dzetysuensis*	PV693698
*Ctenocephalides canis*	NC_063710
*Ctenocephalides felis*	NC_049858
*Ctenocephalides felis felis*	MK941844
*Ctenocephalides felis felis*	MW420044
*Ctenocephalides orientis*	NC_073009
*Ctenophthalmus quadratus*	NC_072692
*Ctenophthalmus yunnanus*	NC_085277
*Dorcadia ioffi*	NC_036066
*Frontopsylla diqingensis*	NC_085276
*Frontopsylla diqingensis*	PP083946
*Frontopsylla elata elata*	PV693697
*Frontopsylla spadix*	NC_073018
*Hystrichopsylla weida qinlingensis*	NC_042380
*Jellisonia amadoi*	NC_022710
*Leptopsylla segnis*	NC_072691
*Macrostylophora euteles*	OR774969
*Neopsylla hongyangensis*	PP133648
*Neopsylla specialis*	NC_073019
*Nosopsyllus laeviceps*	PP838812
*Oropsylla silantiewi*	PX979559
*Palaeopsylla remota*	PQ858441
*Paradoxopsyllus custodis*	OQ627398
*Pulex irritans*	NC_063709
*Stenischia humilis*	NC_073020
*Stenischia montanis*	PP990561
*Stenischia montanis yunlongensis*	OR780663
*Stenoponia polyspina*	OR834393
*Thaumapsylla breviceps orientalis*	PP973737
*Tunga penetrans*	PV426769
*Xenopsylla cheopis*	MW310242
*Boreus elegans*	HQ696579

From the perspective of tree topology, the order Siphonaptera was divided into two primary branches in both trees. One clade was dominated by the family Pulicidae, which formed a well-supported monophyletic clade in both BI and ML trees (BP = 100, PP = 1), and the other clade included four superfamilies (Ceratophylloidea, Vermipsylloidea, Hystrichopsylloidea, and Pygiopsylloidea) as well as the family Hectopsyllidae. Within this framework, Ceratophylloidea formed a relatively stable clade including Ceratophyllidae, Ischnopsyllidae, and Leptopsyllidae, and this superfamily was strongly supported as monophyletic (BP = 100, PP = 1). Remarkably, neither Ceratophyllidae nor Leptopsyllidae was recovered as strictly monophyletic in our analyses. Specifically, *P. custodis* (Leptopsyllidae) was placed within Ceratophyllidae, as the sister group of *M. euteles* (BP = 100, PP = 1).

Within Ceratophyllidae, both analyses recovered broadly similar terminal composition, but differed in internal branching patterns. In addition, some internodes within the family exhibited low to moderate support values, and several closely related lineages were connected by short internodes. In the ML tree, *Amphalius spirataenius* was the first lineage to diverge, followed by *Jellisonia amadoi*, which further split into two major subclades: (i) *O. silantiewi* + (*Macrostylophora euteles* + *Paradoxopsyllus custodis*), and (ii) *Nosopsyllus laeviceps* together with nested relationships among *Ceratophyllus, Callopsylla*, and *Citellophilus* species. In contrast, the BI tree also supported *A. spirataenius* as the earliest diverging lineage, but differed in the placement of *J. amadoi* and *O. silantiewi*, which together formed a basal subclade with *M. euteles* and *P. custodis*. The remaining internal relationships within Ceratophyllidae were largely consistent between the two analyses.

Across both analyses, the two newly sequenced taxa were consistently placed within Ceratophyllidae. *C. dolabris* was associated with the *Citellophilus* lineage in both analyses (BP = 85, PP = 1). *O. silantiewi* showed alternative placements between ML and BI analyses, with moderate bootstrap support in the ML analysis (BP = 61) and high posterior probability in the BI analysis (PP = 0.998).

## 4 Discussion

Consistent with previously reported flea mitogenomes, the two newly sequenced genomes retain the hypothesized ancestral insect arrangement and the conserved 7-bp traditional overlap between atp8 and atp6, a characteristic of arthropod mitogenomes (47, 49). The difference in mitogenome size between the two species is mainly associated with variation in the length of the control region, which may contribute to mitochondrial genome size diversity in Siphonaptera (50). The pronounced A + T bias, negative skewness, and preference for A/U-ending codons observed here are concordant with the general compositional pattern of mitogenomes in Siphonaptera likely shaped by long-term mutational pressure and translational selection (51–56).

Selection pressure analyses indicated that most PCGs in fleas are generally subject to purifying selection, consistent with strong functional constraints on the oxidative phosphorylation system (57). Among all analyzed genes, *atp8* had the highest Ka/Ks ratio and the greatest value dispersion, in agreement with previous mitogenomic studies of fleas (50, 58). This elevated ratio should be interpreted with caution. On one hand, the short length of *atp8* may increase the sensitivity of Ka/Ks estimation to stochastic variation, potentially inflating rate heterogeneity (49, 59). On the other hand, as an accessory subunit of ATP synthase, *atp8* may experience relatively relaxed purifying selection compared with core respiratory chain genes such as *cox1* (57). Taken together, these factors may contribute to the observed pattern of elevated and variable evolutionary rates in *atp8*.

Phylogenetic analyses recovered Ceratophylloidea as a monophyletic superfamily (BP = 100, PP = 1) and Pulicidae as a monophyletic family (BP = 100, PP = 1), consistent with previous phylogenetic analyses (19, 60–62). At the family level, neither Ceratophyllidae nor Leptopsyllidae formed a strictly monophyletic group, with strong support for the nested placement of P. custodis (Leptopsyllidae) within the Ceratophyllidae clade (BP = 100, PP = 1), consistent with a recent mitogenome-based study (63). Discrepancies with multi-locus studies (19, 64) likely stem from variable taxon sampling and marker choice. The two newly sequenced species were both placed within Ceratophyllidae, consistent with previous multi-locus phylogenetic findings (64). However, *O. silantiewi* exhibited inconsistent phylogenetic placement between ML and BI analyses and relatively low nodal support in the ML tree (BP = 61). Such topological instability may be associated with the relatively recent diversification of Ceratophyllidae (65), where close genetic distances and short internodes result in low node support and discordance between different phylogenetic inference methods (66, 67). Moreover, several families such as Ischnopsyllidae, Stivaliidae, Vermipsyllidae, and Hectopsyllidae are currently represented by only one or two available mitogenomes, which may limit the robustness of phylogenetic inference. Therefore, resolving the monophyly or paraphyly of Siphonaptera lineages will require expanded mitochondrial sampling across underrepresented taxa.

## 5 Conclusion

In conclusion, this study presents the complete mitochondrial genomes of *C. dolabris* and *O. silantiewi*, adding new sequence data to the current mitochondrial resources available for Siphonaptera. Phylogenetic analyses based on the expanded dataset provide a general overview of relationships within the group. As our inferences are based exclusively on mitochondrial genomes and limited taxon sampling, the phylogenetic relationships recovered herein should be interpreted with caution and further evaluated using broader taxon and gene sampling. These data enrich mitochondrial genomic resources of Siphonaptera and may facilitate future investigations of flea evolution and phylogenetic relationships.

## Data Availability

The original contributions presented in the study are publicly available. This data can be found here: NCBI repository, accession number is PRJNA1483469.
