# Supplementary material for: New mitogenomic evidence for the phylogeny of Siphonaptera: insights from Callopsylla dolabris and Oropsylla silantiewi
Source: Front Vet Sci. 2026 Aug 4;13:1876060. doi: 10.3389/fvets.2026.1876060 (PMC13481190; doi:10.3389/fvets.2026.1876060)
Supplement: Supplementary file 1 [file Data_Sheet_1.docx]

Supplementary Material

# 1 Supplementary Data

Supplementary data include sequence alignments, partition files, model selection results, and phylogenetic tree files used in this study.

# 2 Supplementary Figures and Tables

## 2.1 Supplementary Figures

**Supplementary Figure S1.** Mitochondrial sequencing depth distribution across the complete mitogenomes of *C. dolabris*. X-axis: genomic position (bp); Y-axis: sequencing coverage (×).

**Supplementary Figure S2.** itochondrial sequencing depth distribution across the complete mitogenomes of *O. silantiewi*. X-axis: genomic position (bp); Y-axis: sequencing coverage (×).

**Supplementary Figure S3.** Combined trace plot of log likelihood (LnL) used to diagnose MCMC convergence of Bayesian phylogenetic analysis, generated from two independent MCMC runs. X-axis: MCMC iteration state; Y-axis: log likelihood values.

**Supplementary Figure S4.** Combined trace plot of log posterior probability (LnPr) used to diagnose MCMC convergence of Bayesian phylogenetic analysis, generated from two independent MCMC runs. X-axis: MCMC iteration state; Y-axis: log posterior probability values.

**Supplementary Figure S5.** Combined trace plot of the gamma shape parameter (α) used to diagnose MCMC convergence of Bayesian phylogenetic analysis, generated from two independent MCMC runs. X-axis: MCMC iteration states; Y-axis: gamma shape parameter values.

**Supplementary Figure S6.** Predicted secondary structures of tRNAs. (A) *C. dolabris* and (B) *O. silantiewi.*

## 2.2 Supplementary Tables

| Species | Family | Superfamily | Accession No. |
| --- | --- | --- | --- |
| *Amphalius spirataenius* | Ceratophyllidae | Ceratophylloidea | OR855715 |
| *Amphipsylla qinghaiensis* | Leptopsyllidae | Ceratophylloidea | PQ571081 |
| *Aviostivalius klossi bispiniformis* | Stivaliidae | Pygiopsylloidea | OR774970 |
| *Aviostivalius klossi bispiniformis* | Stivaliidae | Pygiopsylloidea | PP963728 |
| *Callopsylla dolabris* | Ceratophyllidae | Ceratophylloidea | PX993261 |
| *Ceratophyllus anisus* | Ceratophyllidae | Ceratophylloidea | NC_073017 |
| *Ceratophyllus wui* | Ceratophyllidae | Ceratophylloidea | NC_040301 |
| *Citellophilus tesquorum* | Ceratophyllidae | Ceratophylloidea | PP418872 |
| *Citellophilus tesquorum dzetysuensis* | Ceratophyllidae | Ceratophylloidea | PV693698 |
| *Ctenocephalides canis* | Pulicidae | Pulicoidea | NC_063710 |
| *Ctenocephalides felis* | Pulicidae | Pulicoidea | NC_049858 |
| *Ctenocephalides felis felis* | Pulicidae | Pulicoidea | MK941844 |
| *Ctenocephalides felis felis* | Pulicidae | Pulicoidea | MW420044 |
| *Ctenocephalides orientis* | Pulicidae | Pulicoidea | NC_073009 |
| *Ctenophthalmus quadratus* | Ctenophthalmidae | Hystrichopsylloidea | NC_072692 |
| *Ctenophthalmus yunnanus* | Ctenophthalmidae | Hystrichopsylloidea | NC_085277 |
| *Dorcadia ioffi* | Vermipsyllidae | Vermipsylloidea | NC_036066 |
| *Frontopsylla diqingensis* | Leptopsyllidae | Ceratophylloidea | NC_085276 |
| *Frontopsylla diqingensis* | Leptopsyllidae | Ceratophylloidea | PP083946 |
| *Frontopsylla elata elata* | Leptopsyllidae | Ceratophylloidea | PV693697 |
| *Frontopsylla spadix* | Leptopsyllidae | Ceratophylloidea | NC_073018 |
| *Hystrichopsylla weida qinlingensis* | Hystrichopsyllidae | Hystrichopsylloidea | NC_042380 |
| *Jellisonia amadoi* | Ceratophyllidae | Ceratophylloidea | NC_022710 |
| *Leptopsylla segnis* | Leptopsyllidae | Ceratophylloidea | NC_072691 |
| *Macrostylophora euteles* | Ceratophyllidae | Ceratophylloidea | OR774969 |
| *Neopsylla hongyangensis* | Ctenophthalmidae | Hystrichopsylloidea | PP133648 |
| *Neopsylla specialis* | Ctenophthalmidae | Hystrichopsylloidea | NC_073019 |
| *Nosopsyllus laeviceps* | Ceratophyllidae | Ceratophylloidea | PP838812 |
| *Oropsylla silantiewi* | Ceratophyllidae | Ceratophylloidea | PX979559 |
| *Palaeopsylla remota* | Hystrichopsyllidae | Hystrichopsylloidea | PQ858441 |
| *Paradoxopsyllus custodis* | Leptopsyllidae | Ceratophylloidea | OQ627398 |
| *Pulex irritans* | Pulicidae | Pulicoidea | NC_063709 |
| *Stenischia humilis* | Hystrichopsyllidae | Hystrichopsylloidea | NC_073020 |
| *Stenischia montanis* | Hystrichopsyllidae | Hystrichopsylloidea | PP990561 |
| *Stenischia montanis yunlongensis* | Hystrichopsyllidae | Hystrichopsylloidea | OR780663 |
| *Stenoponia polyspina* | Ctenophthalmidae | Hystrichopsylloidea | OR834393 |
| *Thaumapsylla breviceps orientalis* | Ischnopsyllidae | Ceratophylloidea | PP973737 |
| *Tunga penetrans* | Hectopsyllidae | Pulicoidea | PV426769 |
| *Xenopsylla cheopis* | Pulicidae | Pulicoidea | MW310242 |
| *Boreus elegans* | outgroup | | HQ696579 |

**Supplementary Table S1.** Taxon sampling information for maximum likelihood (ML) and Bayesian (BI) phylogenetic analyses of fleas. Each row presents the full species name, its assigned family, superfamily, and the GenBank accession number used in this study.

| Parameter | ESS | PSRF |
| --- | --- | --- |
| LnL | 2131 |  |
| LnPr | 2529 |  |
| TL{all} | 2520 | 1.002048 |
| r(A<->C){all} | 1743 | 1.000059 |
| r(A<->G){all} | 929 | 0.999671 |
| r(A<->T){all} | 721 | 1.00672 |
| r(C<->G){all} | 1277 | 1.002138 |
| r(C<->T){all} | 1005 | 1.000835 |
| r(G<->T){all} | 1747 | 0.9997922 |
| pi(A){all} | 1149 | 1.000013 |
| pi(C){all} | 852 | 1.004193 |
| pi(G){all} | 717 | 1.002715 |
| pi(T){all} | 1260 | 0.9999821 |
| alpha{all} | 2351 | 1.000422 |
| pinvar{all} | 3343 | 0.9997393 |
| MCMC convergence summary: ASDSF = 0.000905 | | |

**Supplementary Table S2.** Summary of MCMC convergence diagnostic statistics for Bayesian phylogenetic analysis. The table lists all model parameters, corresponding Effective Sample Size (ESS) values, and Potential Scale Reduction Factor (PSRF) values. LnL denotes log likelihood, and LnPr denotes log posterior probability. TL represents total tree length. Substitution rates (r) correspond to nucleotide exchange rates among A, C, G, and T. pi(A/C/G/T) denotes stationary base frequencies, α is the gamma shape parameter, and pinvar represents the proportion of invariable sites. ASDSF refers to the average standard deviation of split frequencies.

| Gene | Position | | | | Size | | Intergenic nucleotides | | Codon | | | | Anti-codon | Strand |
| --- | --- | --- | --- | --- | --- | --- | --- | --- | --- | --- | --- | --- | --- | --- |
|  | From | | To | |  |  |  |  | Start | | Stop | |  |  |
|  | *C. dolabris* | *O. silantiewi* | *C. dolabris* | *O. silantiewi* | *C. dolabris* | *O. silantiewi* | *C. dolabris* | *O. silantiewi* | *C. dolabris* | *O. silantiewi* | *C. dolabris* | *O. silantiewi* |  |  |
| *trnI* | 1 | 1 | 64 | 63 | 64 | 63 | 1 | 3 |  |  |  |  | GAT | J |
| *trnQ* | 66 | 67 | 134 | 135 | 69 | 69 | -1 | 10 |  |  |  |  | TTG | N |
| *trnM* | 134 | 146 | 200 | 212 | 67 | 67 |  |  |  |  |  |  | CAT | J |
| *nad2* | 201 | 213 | 1214 | 1223 | 1014 | 1011 | -2 | 3 | ATT | ATT | TAA | TAA |  | J |
| *trnW* | 1213 | 1227 | 1279 | 1293 | 67 | 67 | 6 | 5 |  |  |  |  | TCA | J |
| *trnC* | 1286 | 1299 | 1346 | 1359 | 61 | 61 |  |  |  |  |  |  | GCA | N |
| *trnY* | 1347 | 1360 | 1410 | 1424 | 64 | 65 | -3 | -3 |  |  |  |  | GTA | N |
| *cox1* | 1408 | 1422 | 2943 | 2957 | 1536 | 1536 | 4 | 4 | ATC | ATC | TAA | TAA |  | J |
| *trnL2* | 2948 | 2962 | 3011 | 3025 | 64 | 64 | 1 | 1 |  |  |  |  | TAA | J |
| *cox2* | 3013 | 3027 | 3693 | 3707 | 681 | 681 | 2 | 2 | ATG | ATG | TAA | TAA |  | J |
| *trnK* | 3696 | 3710 | 3765 | 3779 | 70 | 70 |  |  |  |  |  |  | CTT | J |
| *trnD* | 3766 | 3780 | 3831 | 3843 | 66 | 64 |  |  |  |  |  |  | GTC | J |
| *atp8* | 3832 | 3844 | 3996 | 4008 | 165 | 165 | -7 | -7 | ATT | ATT | TAA | TAA |  | J |
| *atp6* | 3990 | 4002 | 4664 | 4676 | 675 | 675 | -1 | -1 | ATG | ATG | TAA | TAA |  | J |
| *cox3* | 4664 | 4676 | 5446 | 5458 | 783 | 783 |  |  | ATG | ATG | TAA | TAA |  | J |
| *trnG* | 5447 | 5459 | 5509 | 5522 | 63 | 64 | 71 |  |  |  |  |  | TCC | J |
| *nad3* | 5581 | 5523 | 5934 | 5873 | 354 | 351 | 1 | -2 | ATT | ATT | TAA | TAG |  | J |
| *trnA* | 5936 | 5872 | 5999 | 5934 | 64 | 63 | -1 | -1 |  |  |  |  | TGC | J |
| *trnR* | 5999 | 5934 | 6061 | 5995 | 63 | 62 | -3 | -3 |  |  |  |  | TCG | J |
| *trnN* | 6059 | 5993 | 6124 | 6059 | 66 | 67 |  |  |  |  |  |  | GTT | J |
| *trnS1* | 6125 | 6060 | 6192 | 6127 | 68 | 68 |  |  |  |  |  |  | TCT | J |
| *trnE* | 6193 | 6128 | 6256 | 6192 | 64 | 65 | -2 | -2 |  |  |  |  | TTC | J |
| *trnF* | 6255 | 6191 | 6319 | 6255 | 65 | 65 |  | 1 |  |  |  |  | GAA | N |
| *nad5* | 6320 | 6257 | 8036 | 7969 | 1717 | 1713 | 1 | 9 | ATG | ATT | T(AA) | TAA |  | N |
| *trnH* | 8038 | 7979 | 8100 | 8039 | 63 | 61 |  |  |  |  |  |  | GTG | N |
| *nad4* | 8101 | 8040 | 9436 | 9309 | 1336 | 1270 | -7 | 59 | ATG | ATG | T(AA) | T(AA) |  | N |
| *nad4l* | 9430 | 9369 | 9723 | 9662 | 294 | 294 | 2 | 2 | ATG | ATG | TAA | TAA |  | N |
| *trnT* | 9726 | 9665 | 9790 | 9732 | 65 | 68 |  |  |  |  |  |  | TGT | J |
| *trnP* | 9791 | 9733 | 9854 | 9796 | 64 | 64 | 11 | 11 |  |  |  |  | TGG | N |
| *nad6* | 9866 | 9808 | 10,372 | 10,314 | 507 | 507 | -1 | -1 | ATC | ATT | TAA | TAA |  | J |
| *cytb* | 10,372 | 10,314 | 11,511 | 11,453 | 1140 | 1140 | 2 | 2 | ATG | ATG | TAA | TAA |  | J |
| *trnS2* | 11,514 | 11,456 | 11,578 | 11,520 | 65 | 65 | 19 | 19 |  |  |  |  | TGA | J |
| *nad1* | 11,598 | 11,540 | 12,539 | 12,469 | 942 | 930 | 1 | 16 | ATG | ATA | TAG | TAA |  | N |
| *trnL1* | 12,541 | 12,486 | 12,602 | 12,547 | 62 | 62 |  |  |  |  |  |  | TAG | N |
| *rrnL* | 12,603 | 12,548 | 13,900 | 13,847 | 1298 | 1300 |  |  |  |  |  |  |  | N |
| *trnV* | 13,901 | 13,848 | 13,969 | 13,915 | 69 | 68 |  | -2 |  |  |  |  | TAC | N |
| *rrnS* | 13,970 | 13,914 | 14,748 | 14,698 | 779 | 785 |  |  |  |  |  |  |  | N |
| OH | 14,749 | 14,699 | 17,104 | 15,914 | 2356 | 1216 | -1 | -1 |  |  |  |  |  | J |

**Supplementary Table S3.** Summary of mitochondrial genes for *C. dolabris* and *O. silantiewi*. Negative values for intergenic nucleotides denote regions with gene overlap. Incomplete stop codons (T or T(AA)) are presumed to be completed by post-transcriptional polyadenylation.

|  | size | | A | | T | | G | | C | | AT | | GC | | AT-skew | | GC-skew | |
| --- | --- | --- | --- | --- | --- | --- | --- | --- | --- | --- | --- | --- | --- | --- | --- | --- | --- | --- |
|  | *C. dolabris* | *O. silantiewi* | *C. dolabris* | *O. silantiewi* | *C. dolabris* | *O. silantiewi* | *C. dolabris* | *O. silantiewi* | *C. dolabris* | *O. silantiewi* | *C. dolabris* | *O. silantiewi* | *C. dolabris* | *O. silantiewi* | *C. dolabris* | *O. silantiewi* | *C. dolabris* | *O. silantiewi* |
| whole genome | 17,104 | 15,914 | 38.6 | 38.9 | 41.4 | 39.5 | 7.8 | 8.2 | 12.3 | 13.4 | 80 | 78.4 | 20.1 | 21.6 | -0.035 | -0.007 | -0.221 | -0.244 |
| PCGs | 11,142 | 11,055 | 32.5 | 32.7 | 44.4 | 43.6 | 11.9 | 11.9 | 11.2 | 11.8 | 76.9 | 76.3 | 23.1 | 23.7 | -0.154 | -0.142 | 0.032 | 0.008 |
| first codon position | 3714 | 3685 | 34.3 | 34.4 | 37.6 | 37.2 | 17.1 | 17 | 11 | 11.4 | 71.9 | 71.6 | 28.1 | 28.4 | -0.046 | -0.04 | 0.216 | 0.197 |
| second codon position | 3714 | 3685 | 20.8 | 20.8 | 47.5 | 47.7 | 14.1 | 14 | 17.5 | 17.5 | 68.3 | 68.5 | 31.6 | 31.5 | -0.39 | -0.393 | -0.108 | -0.113 |
| third codon position | 3714 | 3685 | 42.4 | 43 | 47.9 | 45.8 | 4.6 | 4.9 | 5.1 | 6.4 | 90.3 | 88.8 | 9.7 | 11.3 | -0.061 | -0.031 | -0.044 | -0.133 |
| tRNAs | 1433 | 1432 | 40.6 | 40.9 | 39.2 | 38.7 | 11.4 | 11.7 | 8.8 | 8.7 | 79.8 | 79.6 | 20.2 | 20.4 | 0.017 | 0.028 | 0.128 | 0.144 |
| rRNAs | 2077 | 2085 | 42 | 41.1 | 39.9 | 40 | 11.8 | 12.9 | 6.2 | 6.1 | 81.9 | 81.1 | 18 | 19 | 0.026 | 0.014 | 0.312 | 0.357 |
| *atp6* | 675 | 675 | 31.7 | 34.1 | 44.6 | 41.3 | 9.8 | 9.6 | 13.9 | 15 | 76.3 | 75.4 | 23.7 | 24.6 | -0.169 | -0.096 | -0.175 | -0.217 |
| *atp8* | 165 | 165 | 37.6 | 38.8 | 44.8 | 47.3 | 4.2 | 3.6 | 13.3 | 10.3 | 82.4 | 86.1 | 17.5 | 13.9 | -0.088 | -0.099 | -0.517 | -0.478 |
| *cox1* | 1536 | 1536 | 28.8 | 29.4 | 41.5 | 39.3 | 14 | 14.5 | 15.7 | 16.9 | 70.3 | 68.7 | 29.7 | 31.4 | -0.181 | -0.144 | -0.057 | -0.079 |
| *cox2* | 681 | 681 | 34.7 | 35.2 | 40.7 | 38.5 | 10.7 | 10.6 | 14 | 15.7 | 75.4 | 73.7 | 24.7 | 26.3 | -0.08 | -0.044 | -0.131 | -0.196 |
| *cox3* | 783 | 783 | 29 | 30.3 | 41.8 | 41.4 | 12.8 | 12.6 | 16.5 | 15.7 | 70.8 | 71.7 | 29.3 | 28.3 | -0.181 | -0.155 | -0.127 | -0.108 |
| *cytb* | 1140 | 1140 | 31.1 | 32.5 | 43.2 | 40.8 | 11.4 | 10.4 | 14.4 | 16.4 | 74.3 | 73.3 | 25.8 | 26.8 | -0.163 | -0.114 | -0.116 | -0.226 |
| *nad1* | 942 | 930 | 32.2 | 31.2 | 45.4 | 45.7 | 15 | 15.8 | 7.4 | 7.3 | 77.6 | 76.9 | 22.4 | 23.1 | -0.171 | -0.189 | 0.336 | 0.367 |
| *nad2* | 1014 | 1011 | 34.1 | 34.1 | 46.7 | 45.3 | 8.6 | 8.8 | 10.6 | 11.8 | 80.8 | 79.4 | 19.2 | 20.6 | -0.156 | -0.141 | -0.103 | -0.144 |
| *nad3* | 354 | 351 | 29.1 | 31.1 | 50.6 | 48.4 | 7.6 | 7.4 | 12.7 | 13.1 | 79.7 | 79.5 | 20.3 | 20.5 | -0.27 | -0.219 | -0.25 | -0.278 |
| *nad4* | 1336 | 1270 | 33.7 | 32.6 | 45.3 | 45.7 | 13.3 | 14.1 | 7.7 | 7.6 | 79 | 78.3 | 21 | 21.7 | -0.147 | -0.167 | 0.267 | 0.297 |
| *nad4L* | 294 | 294 | 37.8 | 37.4 | 46.3 | 46.9 | 12.9 | 13.3 | 3.1 | 2.4 | 84.1 | 84.3 | 16 | 15.7 | -0.101 | -0.113 | 0.617 | 0.696 |
| *nad5* | 1717 | 1713 | 34.6 | 33.4 | 44.5 | 46.5 | 14 | 13.6 | 6.9 | 6.5 | 79.1 | 79.9 | 20.9 | 20.1 | -0.125 | -0.164 | 0.337 | 0.351 |
| *nad6* | 507 | 507 | 35.3 | 36.7 | 49.1 | 47.3 | 5.7 | 4.9 | 9.9 | 11 | 84.4 | 84 | 15.6 | 15.9 | -0.164 | -0.127 | -0.266 | -0.383 |

**Supplementary Table S4.** Nucleotide composition and skewness of the mitochondrial genomes for *C. dolabris* and *O. silantiew*.
